# Complete Genome Sequence of Sinorhizobium meliloti Strain AK21, a Salt-Tolerant Isolate from the Aral Sea Region

**DOI:** 10.1128/MRA.01432-19

**Published:** 2020-01-09

**Authors:** José I. Jiménez-Zurdo, Francisco Martínez-Abarca, José F. Cobo-Díaz, José A. López-Contreras, Manuel Fernández-López, Nicolás Toro

**Affiliations:** a Structure, Dynamics, and Function of Rhizobacterial Genomes Research Group, Department of Soil Microbiology and Symbiotic Systems, Estación Experimental del Zaidín, Consejo Superior de Investigaciones Científicas (CSIC), Granada, Spain; b Departamento de Higiene y Tecnología de los Alimentos, Facultad de Veterinaria, Universidad de León, León, Spain; University of Southern California

## Abstract

We report here the complete genome sequence of the salt-tolerant Sinorhizobium meliloti strain AK21, isolated from nodules of Medicago sativa L. subsp. *ambigua* inhabiting the northern Aral Sea Region. This genome (7.36 Mb) consists of a chromosome and four accessory plasmids, two of which are the symbiotic megaplasmids pSymA and pSymB.

## ANNOUNCEMENT

The mutualistic symbioses established between certain species of the *Alphaproteobacteria* and *Betaproteobacteria* classes (collectively known as rhizobia) and legumes provide a great proportion of the N_2_ in the biosphere (1). Symbiotic nitrogen fixation is compromised by the harsh conditions (e.g., drought or salinity) commonly affecting most agricultural soils (2, 3). Thus, management of legume varieties and rhizobial strains naturally adapted to abiotic stress may improve crop yields in unfavorable environments (4). The widespread interactions between the α-rhizobium Sinorhizobium meliloti and legumes of the Medicago genus are a model experimental system to investigate symbiosis (5–7). The two most recently published S. meliloti draft genome sequences correspond to strains AK170 and AK555, isolated from wild-growing *Medicago* plants in northwest Kazakhstan in the Aral Sea region (8–10). This area suffers from man-made pollution, drought, and salinization, thus constituting a source of salt-tolerant rhizobia with expected increased symbiotic competence under these extreme conditions (11).

We announce here the complete, fully assembled genome sequence of another member of this Aral Sea strain collection, S. meliloti AK21 (SmeAK21), isolated from the nodules of *Medicago sativa* L. subsp. *ambigua* (formerly named Medicago trautvetteri) plants (8). A previous prospective study already unveiled the possession of specific metabolic capabilities by SmeAK21 and large genomic differences with respect to the S. meliloti reference strain Rm1021 (12, 13). The total DNA was obtained with a Real genomic DNA purification kit (Durviz S.L.) from a pure SmeAK21 culture in complete tryptone yeast broth (14). Sequencing was performed on the GS FLX Titanium platform (Roche Diagnostics) at Macrogen, Inc. (South Korea), using both shotgun and 3-kb mate-paired libraries prepared according to Roche 454 standard protocols (15), which delivered totals of 1,607,710 (average length, 719 bp) and 520,432 (average length, 414 bp) reads, respectively. The quality (Phred) score was 40 or above for 99.98% of the bases. The raw data were processed using the Roche GS FLX software (v2.6) and the GS De Novo Assembler tool (v2.6), using default parameters. These analyses assembled the reads into 27 primary scaffolds (*N*_50_ value, 587,174 bp; average length, 266,493 bp; longest scaffold, 1,524,556 bp), predicting 190-fold coverage of the genome. Intra- and interscaffold gaps were further closed by mapping and assembling the specific 3-kb mate-paired library reads to both borders of each gap using the tools of the Geneious Basic platform (16). When required, the Rm1021 genome was used as a reference (12). The complete genome consists of 7.36 Mb distributed into five replicons with the following features (size and G+C content, respectively): the main chromosome (3,785,735 bp and 62.7%), two symbiotic megaplasmids that are present in all S. meliloti strains, i.e., pSymA (1,506,823 bp and 55.7%) and pSymB (1,676,618 bp and 62.4%), and two smaller accessory plasmids that we have termed pSmeAK21a (151,735 bp and 61.5%) and pSmeAK21b (209,149 bp and 59.3%). All four plasmids have recognizable rhizobial *repABC* replication origins (17). Annotation was carried out using the NCBI Prokaryotic Genome Annotation Pipeline, which predicted 6,810 protein-coding genes, 53 tRNA loci, and 3 rRNA operons.

This genome provides a new resource to investigate the adaptive mechanisms of rhizobia to abiotic stress and their potential exploitation in sustainable agricultural practices.

### Data availability.

The sequences of the SmeAK21 chromosome, pSymA, pSymB, pSmeAK21a, and pSmeAK21b have been deposited at GenBank under the accession numbers CP026525, CP026526, CP026527, CP026528, and CP026529, respectively. The Sequence Read Archive (SRA) accession numbers for the raw data are SRR10441808, SRR10441809, and SRR10441810. The BioProject accession number is PRJNA432049.
